# A Preliminary Observational Case Series of Combined Genicular Nerve Block and iPACK for Total Knee Arthroplasty

**DOI:** 10.3390/jcm15041546

**Published:** 2026-02-15

**Authors:** Francesco Tasso, Giuseppe Monteleone, Francesco Marrone, Danilo Esposito, Emilia Cialdella, Marco Minelli, Marco Scrivano, Marco Scardino

**Affiliations:** 1 IRCCS Humanitas Research Hospital, Rozzano, 20089 Milan, Italy; francesco.tasso@humanitas.it (F.T.); giuseppe.monteleone@humanitas.it (G.M.); emilia.cialdella@humanitas.it (E.C.); marcomariaminelli@gmail.com (M.M.);; 2 Department of Anesthesiology and Intensive Care, Santo Spirito Hospital, 00193 Rome, Italy; 3 Department of Biomedical Sciences, Humanitas University, Pieve Emanuele, 20072 Milan, Italy; 4 Department of Orthopaedic Surgery, Sant’Andrea Hospital, “Sapienza” University of Rome, 00189 Rome, Italy; marco.scrivano@uniroma1.it; 5 Department of Orthopaedic Surgery, Santo Spirito Hospital, 00193 Rome, Italy

**Keywords:** total knee arthroplasty, knee replacement, postoperative pain, regional anesthesia, ultrasound-guided nerve block, motor-sparing analgesia, genicular nerve block, iPACK, ERAS, multimodal analgesia

## Abstract

**Background/Objectives:** Total knee arthroplasty (TKA) is associated with severe postoperative pain that can impair early mobilization and prolong recovery. While adductor canal block combined with iPACK is commonly recommended, this strategy may provide incomplete articular coverage and occasionally compromise quadriceps function. Genicular nerve block (GNB) selectively targets the sensory innervation of the knee, and when combined with iPACK may achieve near-complete joint analgesia while preserving motor function. This case series aimed to evaluate the magnitude and duration of analgesia and functional recovery provided by a four-nerve GNB combined with iPACK. **Methods:** Five patients undergoing unilateral TKA under spinal anesthesia received ultrasound-guided blocks of the superolateral, superomedial, inferomedial genicular nerves and the nerve to vastus intermedius, together with an iPACK block. Ropivacaine combined with dexamethasone and dexmedetomidine was used. The primary outcome (pain intensity) [Numeric Rating Scale, NRS] and secondary outcomes (time to first rescue analgesia, opioid consumption, range of motion, and complications) were recorded up to 72 h postoperatively within a standardized multimodal analgesic protocol. **Results:** Pain scores at rest and on movement remained consistently below 3/10 in all patients. Three patients required no rescue analgesia, while two required a single dose at 36–40 h. Knee range of motion exceeded 90° in all cases, and early mobilization was achieved without quadriceps weakness. No neurological or systemic adverse events occurred. **Conclusions:** Four-nerve genicular block combined with iPACK, enhanced with perineural adjuncts, provided prolonged, opioid-sparing analgesia with preserved motor function after TKA. This joint-selective, motor-sparing strategy warrants further evaluation in randomized trials.

## 1. Introduction

Total knee arthroplasty (TKA) is frequently associated with severe postoperative pain, which may impair early mobilization, delay rehabilitation, and prolong hospital stay [1]. Contemporary enhanced recovery after surgery (ERAS) pathways emphasize not only effective analgesia, but also preservation of motor function to facilitate early ambulation and functional recovery. Traditionally, femoral nerve block—and more recently the combination of adductor canal block (ACB) and iPACK (infiltration between the popliteal artery and posterior knee capsule)—has been recommended as a procedure-specific analgesia technique for TKA [2]. These approaches primarily target the infrapatellar branch of the saphenous nerve and the sensory innervation of the posterior capsule and cruciate ligaments. Although ACB was developed as a motor-sparing alternative to femoral nerve block, preservation of quadriceps strength depends on accurate anatomical placement and ultrasound guidance, and clinically relevant weakness has still been reported [3]. In addition, ACB provides limited coverage of posterior knee pain, and emerging concerns regarding ACB-related neuropathy have further stimulated interest in alternative, more anatomically selective approaches [4].

Genicular nerve block (GNB) represents a different analgesic paradigm, selectively targeting the sensory innervation of the knee joint. The superolateral (SLGN), superomedial (SMGN), inferomedial (IMGN) genicular nerves, together with the nerve to vastus intermedius, provide sensory input to the anterior and medial compartments of the knee [5]. While blockade of the nerve to vastus intermedius induces paresis of this specific muscle component, functional knee extension is preserved through the maintained activity of the rectus femoris, vastus lateralis, and vastus medialis, thereby ensuring adequate quadriceps strength for postoperative mobilization. However, because the posterior capsule is primarily innervated by branches of the tibial, obturator, and common peroneal nerves, GNB alone does not provide complete posterior knee analgesia. The iPACK block complements GNB by selectively anesthetizing the terminal sensory branches supplying the posterior capsule without inducing motor weakness. The combination of GNB and iPACK therefore offers a conceptually attractive strategy for near-complete sensory denervation of the knee joint while preserving muscle strength and joint stability.

Emerging evidence supports the combined use of genicular nerve block and iPACK for postoperative analgesia after total knee arthroplasty. A randomized controlled trial comparing GNB alone with GNB combined with iPACK demonstrated that the combined approach significantly reduced pain scores, opioid consumption, and improved early functional recovery within the first 24 h after TKA [6,7]. Additional comparative studies have shown that GNB combined with iPACK provides effective motor-sparing analgesia when compared to adductor canal block-based strategies [8,9], and observational reports have confirmed the clinical feasibility of this combination in real-world practice [10]. Network meta-analyses incorporating multiple peripheral nerve block strategies further suggest that combinations including genicular nerve targets may optimize pain control during early mobilization [11]. However, data regarding the duration of analgesia and functional outcomes beyond the immediate postoperative period remain limited, particularly regarding the role of perineural adjuvants in prolonging block duration.

The present case series explores the clinical performance of a four-nerve GNB combined with iPACK block, both enhanced with perineural dexamethasone and dexmedetomidine, as part of a multimodal analgesic regimen for unilateral TKA performed under spinal anesthesia. Among perineural adjuvants, dexamethasone and dexmedetomidine have demonstrated efficacy in prolonging sensory blockade [12,13]. The primary objective was to assess the magnitude and duration of postoperative analgesia up to 72 h. Secondary objectives included opioid requirements, early mobilization, range of motion (ROM), and block-related complications, thereby extending current evidence beyond the early 24-h window. This work is presented as a descriptive observational case series conducted with a predefined analgesic protocol. Although data collection followed a prospectively planned clinical pathway, no comparator group, hypothesis testing, or inferential statistical analysis was undertaken. The intent was exploratory and hypothesis-generating rather than confirmatory, consistent with established definitions of case series methodology.

This manuscript adheres to the applicable CARE Checklist for Case Series (Supplementary Materials: Table S1).

## 2. Materials and Methods

This was a single-arm, prospective, descriptive uncontrolled observational study including consecutive patients undergoing unilateral primary total knee arthroplasty who received a standardized multimodal analgesic regimen incorporating a four-nerve genicular nerve block combined with iPACK block.

During the preparation of this manuscript/study, the authors used Prism (an AI-assisted LATEX editor) and ChatGPT 5.2 for language checking and refinements. The authors have reviewed and edited the output and take full responsibility for the content of this publication.

### 2.1. Ethical Approval and Informed Consent

Written informed consent was obtained from all the patients for the proposed blocks and rescue strategy. The study was conducted in accordance with the Declaration of Helsinki, and approved by the Institutional Review Board (IRB)/Ethics Committee of Humanitas University (protocol no. 618/17, date of approval 18 December 2017). Peripheral nerve blocks were performed by operators with more of 10 years of experience in regional anesthesia, under sterile conditions after spinal anesthesia.

### 2.2. ERAS-Based Perioperative Protocol

All patients followed a standardized protocol that included: intravenous midazolam (2 mg) before spinal anesthesia, intraoperative fentanyl (50–100 mcg) and/or midazolam (up to 3 mg), as needed, and pre-incision administration of tranexamic acid (2 g), ondansetron (4 mg), metoclopramide (10 mg), and dexamethasone (8 mg). Postoperatively, patients received oxycodone/naloxone 5/2.5 mg per os and a buprenorphine transdermal patch (5 mcg/h) in post-anesthesia care unit (PACU). In ward, paracetamol (1 g every 8 h), ketoprofen (100 mg every 12 h), tizanidine (2 mg at bedtime), metoclopramide (10 mg every 12 h). Rescue analgesia consisted of oxycodone/paracetamol (5 mg/325 mg every 8 h). A second option in the case of failure of GNB would be a rescue ACB, in the case of resting pain NRS > 4. Pain intensity was assessed using the Numeric Rating Scale (NRS) score up to 72 h postoperatively. Early mobilization was attempted within 6–8 h after surgery, and time to first rescue analgesia, range of motion (ROM), and complications were recorded.

Postoperative analgesia was administered according to a standardized ERAS-based multimodal regimen. Non-opioid analgesics (paracetamol and ketoprofen) and a low-dose muscle relaxant (tizanidine) were administered on a scheduled basis to provide baseline analgesia, reduce central sensitization, and minimize opioid requirements. The purpose of the regional anesthesia technique was therefore not to replace systemic analgesia, but to enhance pain control during movement, facilitate early mobilization, and reduce the need for rescue opioids.

### 2.3. Regional Anesthesia Technique

To ensure procedural consistency, all spinal anesthetics and peripheral nerve blocks were performed by a single anesthesiologist with more than 10 years of experience in ultrasound-guided regional anesthesia. Blocks were performed in a fixed anatomical sequence (superolateral, superomedial, inferomedial genicular nerves, nerve to vastus intermedius, followed by iPACK), using standardized patient positioning, identical equipment, and uniform aseptic technique across all cases. All blocks were performed under ultrasound guidance using a high-frequency linear probe (5–10 MHz) and an out-of-plane needle approach, under full sterile conditions. At each injection site, negative aspiration for blood was confirmed prior to injection. A volume of 10 mL of local anesthetic was administered per target, with real-time ultrasound confirmation of appropriate spread within the intended fascial plane.

The four genicular nerve blocks (superolateral, superomedial, inferomedial genicular nerves, and the nerve to vastus intermedius) were each performed using 10 mL of ropivacaine 3.3 mg/mL combined with perineural dexmedetomidine (1 µg/mL) and dexamethasone (0.08 mg/mL). The iPACK block was performed using 15 mL of ropivacaine 2 mg/mL with the same concentrations of dexmedetomidine and dexamethasone. The total injectate per knee was 55 mL, corresponding to a cumulative dose of 160 mg of ropivacaine, 55 µg of dexmedetomidine, and 4.4 mg of dexamethasone.

For the superolateral genicular nerve block (SLGN), the ultrasound probe was positioned in the long axis over the distal lateral femur at the diaphyseal–epiphyseal junction, oriented lateromedially at approximately 90° to the femoral shaft. After identification of the superolateral genicular artery (SLGA), local anesthetic was injected deep to the vastus lateralis muscle, with visualization of separation between the muscle and the periosteum, confirming correct needle placement.

For the superomedial genicular nerve block (SMGN), the probe was placed in the long axis over the distal medial femur at the diaphyseal–epiphyseal junction, oriented mediolaterally at approximately 90° to the femoral shaft. Following identification of the superomedial genicular artery (SMGA), the injection was performed deep to the vastus medialis muscle, confirming separation from the periosteum as evidence of correct spread.

For the inferomedial genicular nerve block (IMGN), the probe was positioned in the long axis over the proximal medial tibia, slightly medial to the tibial tuberosity, and oriented approximately 90° to the tibial shaft. Once the inferomedial genicular artery (IMGA) was identified beneath the medial collateral ligament, local anesthetic was injected between the ligament and the periosteum, with sonographic confirmation of adequate fascial plane spread.

**Figure 1:**
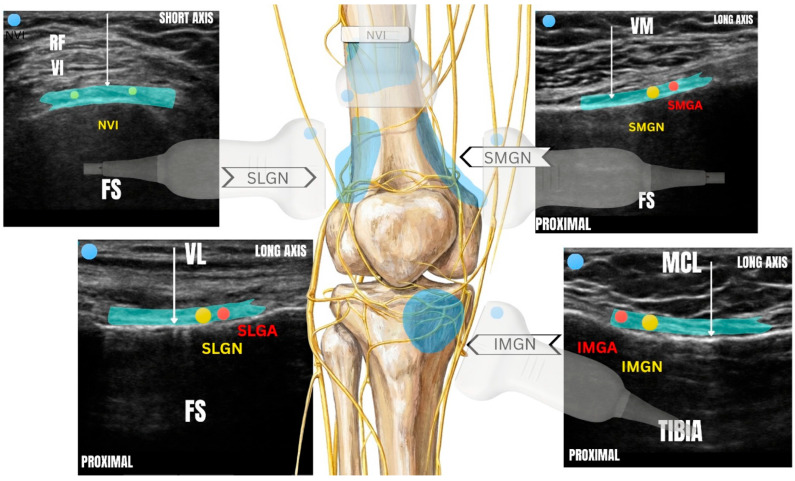
Anatomical representation and ultrasound-relevant anatomy for the four-nerve genicular block.

Finally, the nerve to vastus intermedius was targeted with the probe placed in the short axis over the distal femur, approximately 2–3 cm proximal to the superior border of the patella and oriented perpendicular to the femoral shaft. After gentle bone contact was achieved, local anesthetic was injected deep to the vastus intermedius muscle, confirming separation from the periosteum and appropriate distribution of the injectate (Figure 1).

The four-nerve genicular block. Anatomical depiction and ultrasound images illustrating the anatomical landmarks and target planes for the four-nerve genicular block. Nerve to vastus intermedius block: short-axis view over the distal femur, 2–3 cm proximal to the superior pole of the patella, showing the injection plane deep to the vastus intermedius muscle and superficial to the femoral periosteum. Superomedial genicular nerve block: long-axis view over the distal medial femur showing the superomedial genicular artery and the target plane deep to the vastus medialis muscle. Inferomedial genicular nerve block: long-axis view over the proximal medial tibia demonstrating the inferomedial genicular artery beneath the medial collateral ligament, with the injection plane between the ligament and periosteum. Superolateral genicular nerve block: long-axis view over the distal lateral femur at the diaphyseal– epiphyseal junction, with identification of the superolateral genicular artery and the injection plane deep to the vastus lateralis muscle, adjacent to the periosteum. FS, Femural Shaft; VL, Vastus Lateralis Muscle; VM, Vastus Medialis Muscle; SLGA, Superior Lateral Genicular Artery; SLGN, Superior Lateral Genicular Nerve; SMGA, Superior Medial Genicular Artery; SMGN, Superior Medial Genicular Nerve; MCL, Median Collateral Ligament; IMGA, Inferior Medial Geniculate Artery; IMGN, Inferior Medial Geniculate Nerve; RF, Rectus Femori Muscle; VI: Vastus Intermedius Muscle. White arrows indicate the block target.

### 2.4. Outcomes and Definitions

The primary outcome (pain intensity) and secondary outcomes (time to first rescue analgesia, opioid consumption, range of motion, and complications) were recorded up to 72 h postoperatively within a standardized multimodal analgesic protocol. Assessments were performed at predefined time points: 4, 6, 8, 12, 24, 36, and 72 h postoperatively, with functional evaluation initiated at 6–8 h during physiotherapy sessions. Pain intensity was assessed using the Numeric Rating Scale (NRS) [12] starting 4 h postoperatively, after resolution of spinal anesthesia. Given the standardized use of multimodal analgesia, NRS values reflect overall postoperative pain control within an ERAS framework rather than untreated nociceptive pain. Accordingly, outcomes were interpreted in conjunction with opioid consumption, time to first rescue analgesia, and functional recovery. Resting pain was defined as pain intensity measured with the patient in a supine position without voluntary knee movement [13]. Dynamic pain was defined as pain intensity measured during active knee flexion or assisted ambulation during physiotherapy. Postoperative complications were classified according to the Clavien–Dindo classification [14] to provide a standardized assessment of complication severity and to capture both surgical and anesthesia-related adverse events. Given the exploratory nature of this preliminary case series and the limited sample size, no inferential statistical analyses were performed. Outcomes were evaluated descriptively using predefined clinical measures, including pain intensity scores at rest and during movement, time to first rescue analgesia, opioid requirements, early mobilization, range of motion, and adverse events. The primary focus was on feasibility, consistency, and duration of analgesia rather than statistical comparison or definitive efficacy conclusions.

## 3. Results

### 3.1. Patient Characteristics

The study included five patients undergoing unilateral primary total knee arthroplasty, with heterogeneous demographic and clinical characteristics (Table 1). The mean age was 71.2 years (range 46–87 years), and three of the five patients were female. Mean body weight was 75 kg, with a mean body mass index of 26.4 kg/m^2^ (range 21–33 kg/m^2^). All patients had relevant medical comorbidities, reflected by American Society of Anesthesiologists physical status (ASA-PS) classifications of II or III, including hypertension, diabetes mellitus, chronic obstructive pulmonary disease, ischemic heart disease, chronic kidney disease, and peripheral arterial disease. The mean surgical duration was 84 min (range 52–102 min). All procedures were performed under spinal anesthesia at the L3–L4 interspace using levobupivacaine. Mean estimated intraoperative blood loss was 162 mL (range 100–200 mL), and no major perioperative complications were recorded.

### 3.2. Clinical Outcomes

With regard to primary outcome, pain scores at rest and on movement remained consistently below 3, besides from the patient 1 whose pain score on the 36-h dynamic pain assessment was 6, with minimal supplemental opioid requirement. Three patients required no rescue analgesia within the first 72 h—remarkable for a single-shot block—while the others only received a single dose. One patient developed intolerance to the buprenorphine patch, which was removed at 24 h without loss of analgesia. The time to first rescue analgesia ranged from 36 to 60 h. Functional recovery was consistent across all patients. Active knee extension was preserved in all cases, with no clinically detectable quadriceps weakness during manual muscle testing performed at 6 h postoperatively. Knee range of motion exceeded 90° of flexion in all patients by 24 h, and all patients were able to ambulate with physiotherapy assistance within 8 h of surgery. No patient reported sensory deficits in the distribution of the sciatic or tibial nerves, and no signs of peroneal nerve dysfunction were observed No local anesthetic systemic toxicity was observed. No surgical complications occurred. Clinical outcomes are shown in Table 2.

## 4. Discussion

This case series suggests the potential of combined GNB and iPACK block to provide effective analgesia following unilateral knee arthroplasty up to 72 h postoperatively, preserving quadriceps muscle function that is crucial for knee stability and postoperative early mobilization. Several motor-sparing regional anesthesia strategies have been described for total knee arthroplasty, including combinations of adductor canal block, iPACK, local infiltration analgesia, and genicular-based techniques [2]. The present study was not designed to establish the superiority or unique advantage of one block over another, but rather to explore the feasibility and clinical performance of a joint-targeted, motor-sparing strategy within a standardized multimodal analgesic framework.

The use of perineural adjuncts, including dexamethasone and dexmedetomidine, represents an integral component of this strategy. Accordingly, the prolonged duration of analgesia observed in this series cannot be attributed solely to the anatomical nerve targets, but rather reflects the combined effect of selective sensory blockade and adjunct-enhanced local anesthetic action. The intent of this preliminary study was therefore not to isolate the independent contribution of block anatomy versus adjuvant pharmacology, but to evaluate the overall clinical profile of the combined approach as it is commonly applied in real-world practice.

In this series, pain scores remained consistently low (below 3/10), opioid requirements were minimal, and functional recovery—including knee range of motion and ambulation—was maintained during the first 72 h after surgery. These findings extend emerging randomized evidence supporting genicular based analgesic strategies and suggest that their benefits may persist well beyond the immediate postoperative period.

A randomized controlled trial demonstrated non-inferiority of GNB (five nerves) compared to local infiltration analgesia in terms of pain control, with superior motor function preservation [6]. A recent trial showed how ultrasound-guided combination of iPACK and GNB provided an optimal analgesic regimen for pain relief after TKA under spinal anesthesia in comparison to GNB alone, although for a short follow-up period (24 h) and without functional assessment beyond basic movement [7]. Our four-nerve strategy (intentionally avoiding inferolateral nerve [ILGN] to reduce risk of peroneal nerve injury) showed prolonged analgesic profile, lasting up to 72 h. Notably, none of the patients reported pain in the lateral knee compartment (typically innervated by the ILGN). The addition of perineural adjuncts probably allowed this prolonged analgesia without continuous techniques. These adjuncts have been shown to enhance the duration and quality of peripheral nerve and fascial plane blocks without increasing neurotoxicity when used at appropriate doses. The choice of dexamethasone and dexmedetomidine as adjuncts was based on robust pharmacological and preclinical data [15,16] and growing clinical evidence supporting their efficacy and safety when used at low, standardized doses. Perineural dexamethasone has consistently been shown to prolong sensory block duration and postoperative analgesia through modulation of receptors on nociceptive C fibers, inhibition potassium channel activity, and attenuation of perineural inflammatory responses. Significant prolongation of analgesia and reduction in opioid consumption without evidence of perineural neurotoxicity at doses between 4 and 8 mg. Dexmedetomidine, a highly selective α_2_-adrenergic agonist, enhances regional anesthesia through peripheral mechanisms, including maintaining the hyperpolarized state of cells by inhibiting the activation of the “I_h_ current” in C and A_δ_ fibers, local vasoconstriction delaying anesthetic absorption, and direct suppression of neural transmission [17,18,19]. Perineural administration has been shown to prolong both sensory block duration and analgesia, with most clinical studies using doses in the range of 0.5–1 µg·kg^−1^ or fixed doses of 25–50 µg. Our dosages aligned to these recommendations and evidences (55 µg for dexmedetomidine [<1 µg/Kg, considering four blocks] and 4.4 mg for dexamethasone, also considering four blocks). The total ropivacaine dosage (approximately 160 mg per knee) remained below systemic toxicity thresholds [20]. Future research should focus not only on direct comparisons between genicular nerve-based techniques and established approaches such as ACB combined with iPACK, but also on potential hybrid strategies. Several limitations of this preliminary study should be acknowledged. First, this was a small, single-arm observational case series without a control or comparator group. As such, the findings are exploratory and hypothesis-generating, and no definitive conclusions regarding comparative effectiveness or superiority over other regional anesthesia techniques can be drawn. The limited sample size also precludes inferential statistical analysis and limits generalizability. Conclusions are exploratory and not inferential. All patients received a multimodal analgesia regimen, making it difficult to distinguish the independent contribution of nerve blocks. Pain scores and functional assessments were not performed in a blinded manner, which may introduce assessment bias. Second, all patients received a comprehensive ERAS-based multimodal analgesic regimen, including scheduled non-opioid systemic analgesics. While this approach reflects contemporary clinical practice, it limits the ability to isolate the independent analgesic contribution of the regional anesthesia technique. Accordingly, pain scores should be interpreted as reflecting overall postoperative pain control within a multimodal framework rather than untreated postoperative nociception. Third, perineural adjuncts (dexamethasone and dexmedetomidine) were used in combination with local anesthetic for all blocks. Although these agents are commonly employed to prolong block duration, their use represents a potential confounding factor, and the prolonged analgesic effects observed cannot be attributed solely to the anatomical targets of the genicular nerve block and iPACK. The relative contributions of block anatomy versus pharmacologic enhancement could not be distinguished in the present design. Fourth, all spinal anesthetics and peripheral nerve blocks were performed by a single experienced anesthesiologist. While this likely contributed to procedural consistency and block success, it may limit external validity, as outcomes may vary with operator experience and technique. Finally, functional outcomes and pain assessments were limited to the early postoperative period (up to 72 h), and longer-term outcomes such as sustained functional recovery, rehabilitation milestones, or chronic pain development were not evaluated.

## 5. Conclusions

In this preliminary observational case series, the combination of ultrasound-guided genicular nerve block and iPACK block was associated with effective postoperative analgesia and preservation of quadriceps motor function following total knee arthroplasty. By selectively targeting the articular sensory innervation of both the anterior and posterior knee compartments, this joint-targeted, motor-sparing strategy was integrated successfully within a multimodal ERAS-based analgesic protocol. When combined with perineural dexamethasone and dexmedetomidine, the technique was associated with consistent pain control and low opioid requirements for up to 72 h postoperatively, without neurological or systemic complications. These findings suggest that genicular-based analgesic strategies combined with iPACK merit further investigation as a feasible component of fast-track knee arthroplasty pathways. Larger, randomized controlled studies are required to confirm these preliminary observations, to determine the relative contributions of block anatomy and adjunct pharmacology, and to define the optimal role of genicular nerve-based techniques within procedure-specific postoperative pain management algorithms.

## Figures and Tables

**Table 1:** Patient demographics (COPD: Chronic obstructive pulmonary disease; BMI: Body mass index; ASA-PS: American Society of Anesthesiologists—Physical Status; NRS: Numeric rating scale; SLGN: superolateral genicular nerve; SMGN: superomedial genicular nerve; IMGN: inferomedial genicular nerve; iPACK: infiltration between popliteal artery and posterior knee capsule; dexm: dexmedetomidine; dexa: dexamethasone).

	Patient 1	Patient 2	Patient 3	Patient 4	Patient 5
Age, y	65	74	84	46	87
Gender, M/F	M	F	M	F	F
Weight, Kg/BMI, Kg/m^2^	84/25	82/33	92/31	57/21	60/22
ASA-PS/Comorbidities	3/Hypertension, diabetes	2/COPD, hypertension	3/Hypertension, diabetes, ischemic heart disease	2/hypertension	3/Chronic kidney disease, peripheral arterial disease
Type of surgery/duration, min	TKA/84	TKA/95	TKA/102	TKA/52	TKA/87
Spinal level/drug	L3–L4/levobupivacaine	L3–L4/levobupivacaine	L3–L4/levobupivacaine	L3–L4/levobupivacaine	L3–L4/levobupivacaine
Blood loss (mL)	140	200	180	100	190

**Table 2:** Clinical outcomes (Rescue analgesia: 1, oxycodone/paracetamol 5 mg/325 mg; * Resolution of spinal anesthesia).

	Patient 1	Patient 2	Patient 3	Patient 4	Patient 5
4 h NRS-rest/dynamic	0/0	0/0	0/0	0/0	0/0
6 h * NRS rest/dynamic	<3/<3	<3/<3	<3/<3	<3/<3	<3/<3
8 h NRS rest/dynamic	<3/<3	<3/<3	<3/<3	<3/<3	<3/<3
12 h NRS rest/dynamic	<3/<3	<3/<3	<3/<3	<3/<3	<3/<3
24 h NRS rest/dynamic	<3/<3	<3/<3	<3/<3	<3/<3	<3/<3
36 h NRS rest/dynamic	<3/6	<3/<3	<3/<3	<3/<3	<3/<3
72 h NRS rest/dynamic	<3/<3	<3/<3	<3/<3	<3/<3	<3/<3
First analgesia request, h/dynamic NRS	36/6	40/6	///	///	///
Second analgesia request, h	///	///	///	///	///
Analgesia administered	1	1	///	///	///
ROM > 90°, yes/no	yes	yes	yes	yes	yes
Side effects	///	///	///	///	///
Clavien-Dindo Classification	1	1	1	1	1

## Data Availability

Clinical data is unavailable due to privacy or ethical restrictions.

## Supplementary Materials

The following supporting information can be downloaded at: https://www.mdpi.com/article/10.3390/jcm15041546/s1, Table S1: CARE Checklist for Case Series.

## Abbreviations

The following abbreviations are used in this manuscript:

TKA	Total knee arthroplasty
ERAS	Enhanced recovery after surgery
ACB	Adductor canal block
iPACK	infiltration between Popliteal Artery and posterior Knee Capsule
GNB	Genicular nerve block
SLGN	Supero-lateral Genicular nerve
SMGN	supero-medial Genicular nerve
IMGN	infero-medial Genicular nerve
