# Supplementary material for: A Preliminary Observational Case Series of Combined Genicular Nerve Block and iPACK for Total Knee Arthroplasty
Source: J Clin Med. 2026 Feb 15;15(4):1546. doi: 10.3390/jcm15041546 (PMC12942430; doi:10.3390/jcm15041546)
Supplement: Supplementary file 1 [file jcm-15-01546-s001.zip › jcm-4117126-supplementary.pdf]

CARE Checklist for Case Series

(Adapted from CARE Guidelines for Case Reports)
Manuscript Title: A Preliminary Observational Case Series of Combined Genicular Nerve Block and iPACK for Total Knee Arthroplasty

---

Checklist Items

**Section/Topic**	**Item #**	**Checklist Item Description**	**Reported (Yes/No/NA)**	**Location in Manuscript**
**Title**	1	The area of focus (e.g., Clinical Specialty) and category (e.g., case series) are identified	**Yes**	Title explicitly states "Preliminary Observational Case Series". Clinical specialty: Regional Anesthesia for Orthopedic Surgery
**Keywords**	2	Key elements of the case series in 2-5 keywords	**Yes**	total knee arthroplasty; genicular nerve block; iPACK
**Abstract**	3a	Introduction: What is unique about this case series?	**Yes**	Extended duration of analgesia up to 72h with motor-sparing approach using perineural adjuvants

**Abstract**	3b	Patient Information: Demographics and intervention	**Yes**	5 patients undergoing unilateral TKA, received four-nerve GNB + iPACK with ropivacaine, dexamethasone, dexmedetomidine
**Abstract**	3c	Results: Outcomes and follow-up	**Yes**	Pain scores < 3, minimal opioid use, ROM >90°, no complications. Follow-up to 72 hours
**Abstract**	3d	Conclusion: Main take-away lesson	**Yes**	Motor-sparing, joint-selective strategy provides prolonged analgesia; warrants further evaluation in randomized trials
**Introduction**	4	Brief background summary and rationale for reporting these cases	**Yes**	Section 1: Gap in evidence beyond 24h for GNB+iPACK; need for prolonged analgesia data with functional outcomes

**Patient Information**	5a	De-identified demographic and clinical information	**Yes**	Table 1 (Section 3.1): Age, gender, BMI, ASA-PS, comorbidities for all 5 patients
**Patient Information**	5b	Medical, family, and psychosocial history relevant to the case	**Yes**	Section 3.1 and Table 1: ASA status, comorbidities (hypertension, diabetes, COPD, ischemic heart disease, CKD, PAD)
**Patient Information**	5c	Patient perspective and experiences	**Not applicable**	This is a prospective observational case series focused on objective clinical outcomes. Patient-reported outcome measures were limited to standardized pain intensity ratings (NRS). Formal qualitative patient perspectives were not systematically collected.

**Clinical Findings**	6	Describe relevant physical examination and clinical findings	**Yes**	Section 3.2: Pain scores (NRS at rest/dynamic), motor function (manual muscle testing, preserved quadriceps strength), ROM (>90° flexion by 24h), neurological examination (no sensory deficits, no peroneal dysfunction)
**Timeline**	7	Describe important dates and times in this case	**Yes**	Section 2.4: Predefined timeline with assessments at 4h, 6h, 8h, 12h, 24h, 36h, and 72h postoperatively. Functional evaluation initiated at 6-8h during physiotherapy. Table 2 presents outcomes across all time points.
**Diagnostic Assessment**	8a	Diagnostic methods (e.g., lab tests, imaging)	**Not applicable**	This is an analgesia study; no diagnostic tests required
**Diagnostic Assessment**	8b	Diagnostic challenges	**Not applicable**	No diagnostic challenges relevant to this case series

**Diagnostic Assessment**	8c	Diagnostic reasoning	**Not applicable**	Not relevant for this intervention study
**Therapeutic Intervention**	9a	Types of intervention (e.g., regional technique)	**Yes**	Section 2.3: Four-nerve genicular block (SLGN, SMGN, IMGN, nerve to vastus intermedius) + iPACK block. Ultrasound-guided, out-of-plane needle approach. Detailed anatomical description and Figure 1

**Therapeutic Intervention**	9b	Administration and dosage	**Yes**	Section 2.3: 10 mL ropivacaine 3.3 mg/mL per genicular nerve (4 sites), 15 mL ropivacaine 2 mg/mL for iPACK, combined with dexmedetomidine (1 μg/mL) and dexamethasone (0.08 mg/mL). Total dose per patient: 160 mg ropivacaine, 55 μg dexmedetomidine, 4.4 mg dexamethasone. Section 2.2 describes a complete ERAS-based multimodal regimen.
**Therapeutic Intervention**	9c	Changes in intervention with explanations	**Yes**	Section 2.2: Rescue ACB protocol defined for GNB failure (resting NRS >4). One patient had a buprenorphine patch removed at 24h due to intolerance (Section 3.2). No changes to nerve block technique required.

**Follow-up and Outcomes**	10a	Clinician and patient-assessed outcomes	**Yes**	Section 3.2 and Table 2: NRS pain scores (rest/dynamic), time to first rescue analgesia (36-60h), opioid consumption, ROM, early mobilization (ambulation within 8h)
**Follow-up and Outcomes**	10b	Important follow-up diagnostic and clinical test results	**Yes**	Section 3.2: Motor function assessment with manual muscle testing at 6h showing preserved quadriceps strength and active knee extension. ROM >90° by 24h in all patients. Neurological examination: no sensory deficits in sciatic/tibial distribution, no peroneal nerve dysfunction, no local anesthetic systemic toxicity. Clavien-Dindo Classification Grade 1 for all patients (Table 2).

**Follow-up and Outcomes**	10c	Intervention adherence and tolerability	**Yes**	Section 3.2: All patients received complete nerve block protocol. One patient developed buprenorphine patch intolerance (removed at 24h without loss of analgesia). All other interventions are well tolerated.
**Follow-up and Outcomes**	10d	Adverse and unanticipated events	**Yes**	Section 3.2: No surgical or anesthesia-related complications. No peroneal nerve palsy, no LAST, no neurological deficits. Buprenorphine patch intolerance in one patient (Grade 1 complication).

**Discussion**	11a	Strengths and limitations of managing these cases	**Yes**	Section 4 (Limitations paragraph): Strengths include standardized ERAS protocol, single experienced operator, comprehensive outcome assessment. Limitations: small sample size, no control group, multimodal analgesia confounding, perineural adjuvants as confounding factor, limited external validity, short-term follow-up only (72h).
**Discussion**	11b	Relevant literature and similar reported cases	**Yes**	Section 4: References to RCTs by Fathi et al. [7] (GNB+iPACK vs GNB alone), Cuñat et al. [6] (GNB vs LIA), PROSPECT guidelines [2, 11], ACB literature [3, 4], perineural adjuvant evidence [12, 14]

**Discussion**	11c	Rationale for conclusions	**Yes**	Section 4 and Section 5 (Conclusions): Consistency of analgesic outcomes across all 5 patients, preservation of motor function, safety profile, integration within ERAS framework. Conclusions explicitly acknowledge exploratory nature and need for confirmatory RCTs.

**Discussion**	11d	Primary "take-away" lessons from this case series	**Yes**	Section 4 (new subsection: "Key clinical implications and take-away lessons"): (1) Combined GNB+iPACK is feasible and motor-sparing within ERAS framework, (2) Perineural adjuvants enable prolonged analgesia beyond 72h, (3) Joint-targeted sensory blockade may offer functional advantages, (4) Controlled comparative studies needed to establish definitive role relative to ACB+iPACK.

**Patient Perspective**	12	Patient should share their perspective or experience	**Not applicable**	This is a prospective (not retrospective) observational case series. While patients provided informed consent and participated voluntarily, formal structured interviews or qualitative assessments of patient experiences were not included in the study design. Patient satisfaction was not a predefined outcome measure.
**Informed Consent**	13	Written informed consent obtained from patients	**Yes**	Section 2.1: "Written informed consent was obtained from all the patients for the proposed blocks and rescue strategy." IRB approval obtained prior to enrollment (protocol no. 618/17). Informed Consent Statement confirms written consent for publication.

Additional Information

This case series has been reported in accordance with the CARE guidelines, adapted for the case series format. All identifying information has been removed to protect patient privacy. Written informed consent was obtained from all patients for the regional anesthesia procedures, data collection, and publication of anonymized data. The study protocol received full Institutional Review Board approval prior to patient enrollment (IRB protocol no. 618/17).
